# Potential value of tripartite motif-containing 59 as a biomarker for predicting the prognosis of patients with lung cancer: A protocol for systematic review and meta-analysis

**DOI:** 10.1097/MD.0000000000026868

**Published:** 2021-08-13

**Authors:** Jianfei Guo, Ke Min, Lichun Deng

**Affiliations:** a Department of Thoracic Surgery, Xingtai People's Hospital of Hebei Medical University, Xingtai, Hebei Province, China; b Department of Cardiothoracic Surgery, Jiujiang First People's Hospital, Jiujiang, Jiangxi Province, China; c Department of Oncology, Jiangyin People's Hospital, Jiangyin, Jiangsu Province, China.

**Keywords:** bioinformatics, lung cancer, meta-analysis, prognosis, protocol, tripartite motif-containing 59

## Abstract

**Background:**

In recent years, related studies have revealed that tripartite motif-containing 59 (TRIM59) is related to the prognosis of lung cancer. However, these results have not been proved by any evidence. Therefore, this study evaluated the relationship between TRIM59 and the prognosis of lung cancer by carrying out meta-analysis. In addition, we explored the mechanism and related pathways of TRIM59 in lung cancer through bioinformatics analysis.

**Methods:**

Comprehensive literature search was performed in China National Knowledge Infrastructure, Chinese Biomedical literature Database, Chinese Scientific and Journal Database, Wan Fang, Web of Science, PubMed, and EMBASE databases, and eligible studies were obtained based on the inclusion and exclusion criteria. The pooled hazard ratios and odds ratios were applied to assess the clinical value of TRIM59 expression for overall survival and clinicopathological features. Meanwhile, meta-analysis was conducted on the Stata 16.0. The mRNA expression level of TRIM59 in lung cancer was analyzed using Oncomine and Gene Expression Profiling Interactive Analysis (GEPIA) database. Gene Set Enrichment Analysis (GSEA) was used to predict the signaling pathways that TRIM59 might be involved in. The correlation between the expression level of TRIM59 in lung cancer and the abundance of immune cell invasion was analyzed by TIMER database. The survival analysis was verified by Kaplan–Meier Plotter database.

**Results:**

The results of this meta-analysis would be submitted to peer-reviewed journals for publication.

**Conclusion:**

In this study, the application of meta-analysis and bioinformatics analysis will provide evidence support for the study on the prognosis and mechanism of TRIM59 in lung cancer.

## 1 Introduction

The incidence and mortality of lung cancer are the highest among malignant tumors, posing a great threat to human health, most of which are non-small cell lung cancer.^[1,2]^ At present, the main treatment methods for lung cancer patients^[3,4]^ include surgical resection, radiotherapy, chemotherapy, targeted therapy and immunotherapy. However, these treatments have their limitations and risk of cancer recurrence. How to scientifically and effectively treat lung cancer is still a great challenge facing the medical community. The occurrence and development of lung cancer is closely related to the abnormal gene expression and signaling pathway.^[5]^

Tripartite motif-containing 59 (TRIM59) is a new member of the tripartite motif family, and it is associated with a variety of tumors.^[6,7]^ Studies have proved that TRIM59, as an oncogene, is up-regulated in lung cancer, stomach cancer, liver cancer, colorectal cancer and other tumors.^[8–10]^ Moreover, TRIM59 can promote the proliferation and migration of tumor cells through a variety of pathways.^[6,11–13]^ TRIM59 is expected to be a new target for tumor diagnosis and treatment.

However, the expression of TRIM59 and its function in lung cancer are still poorly understood. Therefore, in this study, bioinformatics analysis and meta-analysis were used to explore the expression and potential function of TRIM59 in lung cancer, so as to provide reference for relevant basic experiments and clinical translational studies.

## 2 Methods

### 2.1 Study registration

This meta-analysis protocol is based on the Preferred Reporting Items for Systematic Reviews and Meta-analysis Protocols (PRISMA-P) statement guidelines.^[14]^ The PRISMA-P was registered on Open Science Framework, and the registration number is DOI 10.17605/OSF.IO/ZTGSE.

### 2.2 Data sources and retrieval strategy

A systematic literature search was conducted through July 2021 to identify relevant articles that are included in this meta-analysis and the search was carried out in the following databases: China National Knowledge Infrastructure, Chinese Biomedical literature Database, Chinese Scientific and Journal Database, Wan Fang, Web of Science, PubMed, and EMBASE databases. The detailed search strategies are listed in Table 1.

**Table 1:** Search strategy in PubMed database.

Number	Search terms
#1	Lung Neoplasms[MeSH]
#2	Cancer of Lung[Title/Abstract]
#3	Lung Cancer[Title/Abstract]
#4	Pulmonary Cancer[Title/Abstract]
#5	Pulmonary Neoplasms[Title/Abstract]
#6	Cancer of the Lung[Title/Abstract]
#7	Neoplasms, Lung[Title/Abstract]
#8	Neoplasms, Pulmonary[Title/Abstract]
#9	Cancer, Lung[Title/Abstract]
#10	Cancer, Pulmonary[Title/Abstract]
#11	Cancers, Lung[Title/Abstract]
#12	Cancers, Pulmonary[Title/Abstract]
#13	Lung Cancers[Title/Abstract]
#14	Lung Neoplasm[Title/Abstract]
#15	Neoplasm, Lung[Title/Abstract]
#16	Neoplasm, Pulmonary[Title/Abstract]
#17	Pulmonary Cancers[Title/Abstract]
#18	Pulmonary Neoplasm[Title/Abstract]
#19	Carcinoma, Non-Small-Cell Lung[MeSH]
#20	Carcinoma, Non-Small Cell Lung[Title/Abstract]
#21	Non-Small Cell Lung Cancer[Title/Abstract]
#22	Non-Small-Cell Lung Carcinoma[Title/Abstract]
#23	Non-Small-Cell Lung Cancer[Title/Abstract]
#24	Carcinoma, Non-Small-Cell Lung[Title/Abstract]
#25	Carcinomas, Non-Small-Cell Lung[Title/Abstract]
#26	Lung Carcinoma, Non-Small-Cell[Title/Abstract]
#27	Lung Carcinomas, Non-Small-Cell[Title/Abstract]
#28	Non-Small-Cell Lung Carcinoma[Title/Abstract]
#29	Non-Small-Cell Lung Carcinomas[Title/Abstract]
#30	Small Cell Lung Carcinoma[MeSH]
#31	Oat Cell Carcinoma of Lung[Title/Abstract]
#32	Carcinoma, Small Cell Lung[Title/Abstract]
#33	Oat Cell Lung Cancer[Title/Abstract]
#34	Small Cell Cancer Of The Lung[Title/Abstract]
#35	Small Cell Lung Cancer[Title/Abstract]
#36	or1-35
#37	Tripartite motif-containing 59[Title/Abstract]
#38	TRIM59[Title/Abstract]
#39	or/37-38
#40	Prognos∗[Title/Abstract]
#41	Survival[Title/Abstract]
#42	or/40-41
#43	#36 and #39 and #42

### 2.3 Inclusion criteria for study selection

The inclusion criteria are as follows: All articles that describe the relationship between TRIM59 expression and survival outcome or clinicopathological parameters in patients with lung cancer; adequate data that are provided to calculate the odds ratio, hazard ratio (HR), and 95% confidence interval (CI); the expression of TRIM59 in lung cancer tissues that are determined by immunohistochemistry.

The exclusion criteria are as follows: the case report, review, letter, and conference abstract; articles describing overlapping studies; or studies with insufficient data.

### 2.4 Data collection and analysis

**Figure 1:**
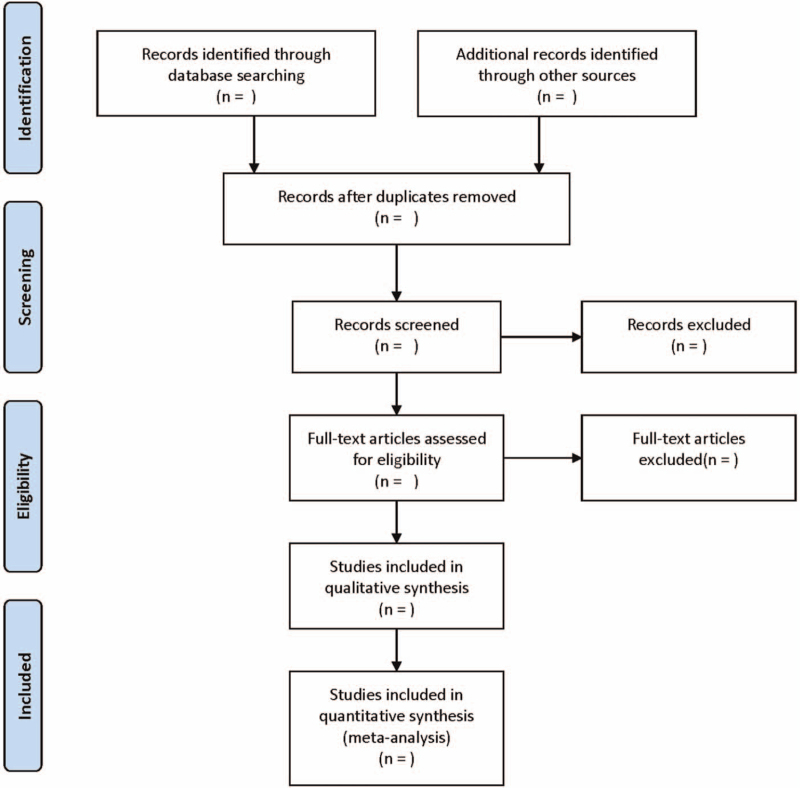
Flow diagram of study selection process.

The literature screening process is displayed in Figure 1. The abstract and title were first read independently by 2 researchers. According to inclusion criteria and exclusion criteria, preliminary screening was conducted, and the included literatures were finally sorted out. The following data were obtained from each included study: the first author, publication year, country, ethnicity, sample size, detection method, clinicopathological parameters, the longest follow-up period, HR, and corresponding 95% CI. If the required data were not provided directly but as Kaplan–Meier curves, it is necessary to use Engauge Digitizer4.1 version to extract HR and its 95% CI from graphic survival curves.^[15,16]^

### 2.5 Quality assessment

The Newcastle–Ottawa Scale (NOS) was used to perform the quality assessment of each included study.^[17]^ NOS score ≥6 indicates that the quality of the literature is high.^[18]^

### 2.6 Measures of prognosis

Overall survival was taken as prognostic outcomes, and the results were expressed as HRs with 95% CIs.

### 2.7 Management of missing data

If there exists insufficient or missing data in the literature, we would only analyze the currently available data and discuss its potential value.

### 2.8 Statistical analysis

Meta-analysis was conducted on the Stata 16.0 (Stata Corporation, TX). HR and its 95% CIs were used to evaluate the relationship between TRIM59 expression and prognosis in patients with lung cancer. Pooled odds ratio and corresponding 95% CI were used to evaluate the association between TRIM59 expression and clinicopathological parameters in lung cancer patients. Cochran's *Q* (chi-square) test and I2 statistics were applied to assess the heterogeneity among the studies. If *P* < .1 and *I*^2^ > 50% were considered as significant heterogeneity, the random-effects model was used or the fixed-effects model was used. *P* values in this study were 2-sided, and *P* < .05 indicated that there were statistical significances.

### 2.9 Additional analysis

#### 2.9.1 Subgroup analysis

According to the detection methods, ethnicity, and the source of survival data, we conducted the subgroup.

#### 2.9.2 Sensitivity analysis

Sensitivity analysis was performed via sequential deletion of a single included study to test.

#### 2.9.3 Reporting bias

Begg's test and Egger's test were carried out to evaluate the potential publication bias.^[19,20]^

## 3 Bioinformatics analysis

The mRNA expression levels of TRIM59 in lung cancer and paracancerous tissues were analyzed using Oncomine database (https://www.oncomine.org/resource/main.html) and Gene Expression Profiling Interactive Analysis (GEPIA) database (http://gepia.cancer-pku.cn/). All lung cancer specimens with TRIM59 gene were selected from Kaplan–Meier plotter database for survival correlation analysis. RNASeq data of TRIM59 gene in lung cancer samples from The Cancer Genome Atlas (TCGA) database (https://www.cancer.gov/about-nci/organization/ccg/research/structural-genomics/tcga) were selected for Gene Set Enrichment Analysis (GSEA). The relationship between TRIM59 expression level and immune cell infiltration in lung cancer was analyzed by TIMER database.

## 4 Ethics

Our research data were derived from published literatures, because there were no patient recruitment and personal information collection. Therefore, ethical approval was not required.

## 5 Discussion

TRIM59 is located on human chromosome 3 and is a member of the C-XI subfamily.^[21]^ Apart from inheriting a highly conserved 3-segment domain, a TM domain is also involved in protein transmembrane localization at its C-terminal that is involved in the localization of TRIM59. TRIM59 protein is located in the endoplasmic omentum of cells and contains 403 amino acid residues. By mediating the interactions between proteins, the ubiquitination regulates the stability of the protein and promotes the disordered proliferation of tumor cells.^[22]^ TRIM59 plays a negative role in the regulation of immune signals through the activation of NF-κB and IRF3/IRF7 mediated signaling pathways.^[23]^ TRIM59 participates in SV40 Tag /p53/PRB and Ras/ERK/PI3K/Akt oncogenic signaling pathways, regulates DNA synthesis in the S phase of the cell cycle, promotes cell division and proliferation, simultaneously upregulates the expression of cell cycle-related proteins, and ultimately induces tumor formation.^[24]^

TRIM59 is not only involved in the molecular mechanism of lung cancer, but is also closely correlated to the clinicopathological characteristics and prognosis of lung cancer.^[25–29]^ The increased expression of TRIM59 indicates a poor clinical prognosis in patients with lung cancer, and may be used as a biomarker to evaluate the prognosis of patients suffering from lung cancer. In this study, the role of TRIM59 in the prognosis of lung cancer patients was analyzed through meta-analysis and bioinformatics.

## Author contributions

**Conceptualization:** Lichun Deng, Jianfei Guo.

**Data curation:** Jianfei Guo.

**Formal analysis:** Jianfei Guo.

**Funding acquisition:** Lichun Deng.

**Methodology:** Ke Min.

**Project administration:** Lichun Deng.

**Resources:** Ke Min.

**Supervision:** Lichun Deng.

**Validation:** Ke Min.

**Writing – original draft:** Lichun Deng, Jianfei Guo, Ke Min.

**Writing – review & editing:** Lichun Deng, Jianfei Guo, Ke Min.
